# A History of Medical Education

**Published:** 1893-03

**Authors:** 


*A History of Medical Education.* By Dr. THEODOR PUSCHMANN. Translated and Edited by EVAN H. HARE. Pp. xi., 650. London: H. K. LEWIS. 1891.

This book should find a place in every central library. It deals very fully with the progress of medical knowledge, side by side with the development of general intellectual culture. It is a full epitome of very much wider study, as indicated by the numerous references to authors of all countries and of all ages. The book is divided into four periods of medical teaching: 1, in ancient times; 2, in the middle ages; 3, in recent times, *i.e.* from the sixteenth century; 4, in the nineteenth century.

In India, as shown by their oldest writings (Vedas, 600 B.C.), a separate medical caste existed from early times, and in the writings of Charaka and Susruta advice was given which might well serve for rules of ethics in the present day. The Indian doctors were excellent surgeons, and used varied and well-designed instruments. The Buddhist monks considered life worthless, and therefore paid no attention to maintaining it, nor cared for remedies in sickness. Yet a Buddhist king started hospitals, which existed in Ceylon 400 B.C. In Egypt the schools of learning taught medicine with other branches of knowledge. Among the Jews medical art was largely associated with the priesthood, and medical officers of health were appointed to all the large towns. The Greeks, before the time of Hippocrates, worshipped Asclepius, the god of the healing art, and superstitious rites were carried out in temples of health by priest doctors, called Asclepiadæ. Hippocrates, 460–377 B.C., studied at Athens in the period of its greatest grandeur, coeval with Pericles, Socrates, and Plato. He applied the Platonic method to medical teaching, to both its science and ethics. Dissections were made on animals, but physiology consisted of unfounded hypotheses and unsupported speculations. Physical diagnosis was of a high order. Alexander of Macedon, 300 B.C., advanced medicine, together with all forms of civilisation and knowledge; and under the Ptolemies two medical schools arose under Herophilus and Erasistratus. Plenty of subjects were found them for dissection, even living criminals. A fine medical education was also given in Pergamus, and here Galen, 131 A.D., received his training.

Coming to the middle ages, Christianity concerned itself only with the moral culture of mankind; to the training of the intellect it remained indifferent. On the other hand, the Church initiated the foundation of hospitals and other benevolent institutions. The devotion of the Christians to the sick and helpless became in later times overshadowed by ignorance and superstition: the power of prayer replaced the help of the doctor. The sufferers came to the church (as they formerly did to the temples of Asclepius) to request advice and aid from the priests. Amulets and miracles henceforth played a prominent part in the medicine of the Christians. Meanwhile the Arabian power had been growing, and in the twelfth century, while much of Europe was steeped in superstition, ignorance, and barbarism, an intellectual life was unfolding on the Spanish peninsula, rich and fruitful in every path of mental activity. In the earliest period of Islam care was devoted to the study of medicine, but unlimited belief in authority interfered with independent investigation, though much was written on the past history of the science. The teachings of Galen in anatomy and physiology were followed almost without question. In practical medicine some slow progress was made, especially in study of exanthemata, but surgery actually lost ground. The cautery was most freely used for all sorts of conditions.

In Baghdad, as early as the ninth century, there existed a hospital with a medical school; and many others are mentioned, among them the splendidly equipped Mansurian hospital at Cairo.

The Mohammedans saw in the hallucinations of lunatics manifestations of a supernatural world, and gave the patients every consideration: whilst in Christendom they were regarded as the prey of evil spirits, and were treated as wizards and witches.

The Arabs introduced apothecaries' shops, and the beginnings of the system of medical examination are probably to be sought for among the Arabs. The Arab doctors seem to have been practical and straightforward. In the *Doctor's Guide* it is said: "The majority of diseases are cured by the help of Nature, without the aid of the doctor." "If you can cure the patient by

5

dietetic means, forbear to order drugs." "Never speak unfavourably of other doctors, for everyone has his successful and his unsuccessful times." "Visit your patient when he is at his worst; at that time come to an understanding with him about your fee. Common people, when they are cured, hate you if they think of the fee." The higher teachings of the Arabs lasted till the fourteenth century.

The quotations given indicate the scope of the work, which is rendered into excellent English by Mr. Hare. The book is most pleasant reading; it is a complete work of reference for the subjects it treats, and it will always take rank as a medical classic, and as a monument to the energy and ability of its talented author.

